# Influential factors related to feeding disorders in preterm infants and the construction of predictive models

**DOI:** 10.3389/fped.2025.1562778

**Published:** 2025-05-14

**Authors:** Lishan Chen, Huichang Zhou, Zhiming Tang, Haiyin Deng, Zhihao Li

**Affiliations:** ^1^ Department of Epidemiology, School of Public Health, Southern Medical University, Guangzhou City, China; ^2^ Department of Rehabilitation Medicine, The First People’s Hospital of Foshan City, Foshan City, China

**Keywords:** preterm infants, feeding disorders, influencing factors, predictive modeling, ROC curves

## Abstract

**Objective:**

To investigate the influencing factors associated with feeding disorders in preterm infants and to construct a prediction model.

**Methods:**

314 cases of preterm infants admitted to our hospital from January 2019 to December 2022 were retrospectively analyzed and divided into feeding disorder group and non-feeding disorder group according to the presence of feeding disorder at 37 weeks of corrected gestational age. Statistical analysis of children's general information, hospitalization measures, laboratory tests, feeding time, etc. Multifactorial Logistic regression analysis of the occurrence of feeding disorders related to the influence of factors, and the use of subjects to make a work characteristic curve to analyze the predictive value of the relevant factors on feeding disorders.

**Results:**

Multifactorial logistic regression analysis suggested that lower birth gestational age, birth weight, white blood cell count, absolute value of monocytes, blood calcium value, Apgar score at 1 min after birth, and longer duration of noninvasive ventilation were risk factors for feeding disorders in preterm infants. ROC curve analysis suggested that the area under the curve of the feeding disorders was predicted by the combination of the above seven indexes to construct the feeding disorders prediction model The AUC was 0.866 (*P* < 0.001, 95% CI 0.801–0.932), and it had a maximum Yoden index of 0.699, an optimal cutoff value of 0.169, a sensitivity of 85.4%, a specificity of 84.5%, and a prediction accuracy of 91.4%.

**Conclusions:**

Lower birth gestational age, birth weight, white blood cell count, absolute monocyte value, blood calcium value, low Apgar score at 1 min after birth, and prolonged noninvasive ventilation are risk factors for feeding disorders in preterm infants, and the present prediction model is a good predictor of the occurrence of feeding disorders in preterm infants.

## Introduction

Preterm labor (births at a gestational age of <37 weeks) (1) is an important issue for neonatal health worldwide. According to the World Health Organization, approximately 15 million preterm babies are born globally each year (2), and the preterm birth rate is approximately 11% and continues to increase (3). Preterm birth is the leading cause of death in children under 5 years of age (4), and its associated complications have a particular impact on quality of survival.

The universal global standard for discharge from the neonatal intensive care unit (5) is the infant's ability to take nutrition orally, known as full oral feeding (FOF) (6). Feeding disorders can affect the process of full oral feeding. Feeding Disorders are defined as the inability of a child to complete oral intake on his or her own, dependence on intravenous nutrition or gastrostomy tube feeding, or a single oral feeding lasting longer than 30 min (7).

The incidence of feeding disorders in preterm infants is as high as 82% due to immature development of sucking-swallowing-breathing coordination (8, 9), which is significantly higher than that of term infants. Such children are prone to gastrostomy tube dependence, leading to reduced tongue muscle activity (10), which in turn triggers degradation of swallowing function, delayed oral transportation and lagged pharyngeal phase initiation, increasing the risk of aspiration pneumonia (11). Both feeding intolerance and feeding disorders can lead to gastrostomy tube dependence, but most of the existing studies focus on the pathomechanisms of feeding intolerance (12, 13), neglecting the feeding disorders caused by deficiencies in the ability to feed by mouth. Feeding disorders not only prolong the duration of gastrostomy tube retention and interfere with the maturation of gastrointestinal dynamics, but also may lead to prolonged hospitalization and increased healthcare costs (12, 13), and even affect the neurodevelopment and social cognitive ability of children (14), which may be life-threatening in severe cases.

Despite the prevalence and consequences of preterm infant feeding problems globally, there are no harmonized guidelines as the gold standard of care (9). Due to the complexity of the mechanism of oral feeding and the immaturity of the preterm infant's organism leading to large individual differences at different months of age, it is difficult to assess the oral feeding function of preterm infants at small months of age. Most of the commonly used methods of assessment are based on scale assessment, which has a certain degree of subjectivity, while the methods used for the objective assessment of preterm infants are limited, and swallowing imaging and swallowing laryngoscopy are not applicable to a wide range of applications due to specific requirements. The smart pacifier system studied by Akbarzadeh (15) and others is expensive equipment and has some limitations in clinical application in preterm infants. Therefore, the construction of a prediction model for feeding disorders in preterm infants may be a new idea to solve the above clinical problems. Due to the fact that there are fewer influencing factors analyzed for feeding disorders in preterm infants, there are no reports on the prediction of feeding disorders model for preterm infants so far. In most studies, low birth gestational age, low birth weight, and concomitant etiologies associated with preterm labor are factors that influence infant feeding and swallowing (16–18). Pre-discharge or perinatal brain injury has not been identified as one of the risk factors for persistent feeding disorders after discharge (19).

To this end, we determined that there is a need to explore relevant evidence to support good predictive modeling to guide clinicians in the early identification of preterm infants with a poor prognosis for feeding disorders, integrating early interventions to increase rehabilitative therapies, as well as to inform caregivers in providing a supportive approach to feeding.

## Hypothesis and objectives

We hypothesized that the maturity of the preterm infant, the degree of perinatal damage to the infant, and the organism's function would be related to the development of the preterm infant's feeding function.

The aim of this study is to investigate the influencing factors of feeding disorders in preterm infants and to construct a prediction model of feeding disorders in preterm infants, so as to provide a scientific basis for further shortening the gastric tube retention time.

### Secondary aims

• Analyze factors related to the development of feeding function in premature infants.
• Evaluate oral feeding function:
  ○ Whether there is dependence on gastric tube.
  ○ Correct the ability to eat orally at a gestational age of 37 weeks.
  ○ Safe oral intake.

## Methods

### Infant recruitment

Retrospective analysis of 314 cases of preterm infants treated in the neonatology department of the First People's Hospital of Foshan City during the period of January 2019-December 2022 were divided into the feeding obstacle group (*n* = 50) and the non-feeding obstacle group (*n* = 264) according to the presence or absence of feeding obstacles at the time of correcting the gestational age of 37 weeks of the child. Inclusion criteria: (1) neonates born at a gestational age of <37 weeks. (2) complete clinical data. Exclusion criteria: (1) Combination of diseases requiring surgery and fasting. (2) necrotizing enterocolitis (NEC). (3) gastrointestinal malformations. (4) serious respiratory tract infections. This study was reviewed by the Ethics Committee of the First People's Hospital of Foshan City [Lun Audit Research (2024) No. 79].

### Methodology

Gather clinically relevant information about the child by reviewing medical records and test results:

(1) General information: gestational age at birth, birth weight, gender, mode of delivery, whether multiple births, length of hospitalization, Apgar score. (2) Hospitalization measures: time of invasive ventilation, non-invasive ventilation, high-flow oxygen, central oxygen. (3) Laboratory tests: serum albumin, serum protein, direct bilirubin, indirect bilirubin, serum calcium, calcitonin, basophil absolute value, eosinophils absolute value, lymphocyte count, red blood cell count, white blood cell count, monocyte count, platelet count, interleukin-6. (4) Feeding time: months after the start of oral feeding Absolute value of basophils, absolute value of eosinophils, lymphocyte count, red blood cell count, white blood cell count, monocyte count, platelet count, interleukin-6. (5) Feeding time: the age of the month of the start of oral feeding, the time of removal of the gastric tube.

### Statistical analysis

SPSS25.0 software was used for statistical analysis. Measurement data were tested for data normality by the Kolmogorov–Smirnov method, and data that conformed to normal distribution were described by mean ± standard deviation, and independent samples t-test was performed; data that did not conform to normal distribution were expressed as median (interquartile spacing) [M ± (Q1, Q3)], and Mann–Whitney *U* test was performed. Count data were expressed as actual number of cases with percentages, and the *χ*² test or Fisher's exact probability method test was performed. Factors associated with feeding disorders in preterm infants were analyzed using univariate analysis, and binary logistic multifactorial regression analysis was used to screen the risk factors and protective factors for the emergence of feeding disorders in preterm infants at corrected gestational age of 37 weeks, and to assess the risk factors and protective factors for the development of feeding disorders in preterm infants at corrected gestational age of 37 weeks by using a receiver operating characteristic (ROC) curve made by the subjects, and assessing the risk factors and protective factors with the area under the curve (area under curve (AUC) was used to assess the ability of the relevant factors to predict feeding disorders, and the critical value was set to the value corresponding to the maximum Jordon index (sensitivity + specificity - 1). All statistical tests were performed with *P* < 0.05 as the difference being significant.

## Results

### Analysis of the distribution of clinical characteristics of preterm infants and baseline values

The gestational age of 314 preterm infants was (33.92 ± 2.85) weeks; birth weight was (2.22 ± 0.71) kg. 314 preterm infants corrected the incidence of feeding disorders at a gestational age of up to 37 weeks was 15.92% (50/314), and the time of gastrostomy tube removal was (36.26 ± 1.04) weeks, with a minimum of 34^+1^ weeks and a maximum of 42^+3^ weeks. The results showed that the incidence of feeding disorders was higher (*P* < 0.05) for lower birth weight, lower gestational age at birth, history of cesarean section, application of invasive positive pressure ventilation, and duration of noninvasive ventilation. There was no significant difference in the gestational age and gender of the children in both groups (*P* > 0.05) (Tables 1, 2).

**Table 1:** Distribution of clinical characteristics of preterm infants.

Sports event	Number of examples	Number of cases of feeding disorders (%)	*χ*^2^ value	*P*-value	Gastric tube removal time [M(Q1, Q3), weeks]	*H/Z* value	*P*-value
Gestational age at birth (weeks)							
≤28	12	6 (50.00%)	20.83	<0.001	37.45 (37.23, 38.43)	80.35	<0.001
28^+1^–32	75	17 (22.67%)			36.60 (36.20, 37.60)		
32^+1^–34	30	8 (26.67%)			35.60 (35.4, 36.53)		
34^+1^–37	197	19 (9.64%)			36.10 (35.40, 36.40)		
Birth weight (kg)							
≤1	13	5 (38.46%)	29.45	<0.001	37.50 (36.85, 38)	78.06	<0.001
1–1.5	60	21 (35%)			37.10 (36.43, 38)		
1.5< × <2.5	84	12 (14.29%)			36.20 (35.5, 36.50)		
>2.5	157	12 (7.64%)			36.10 (35.40, 36.40)		
Distinguishing between the sexes							
Male	179	29 (16.20%)	0.024	0.88	36.30 (35.50, 36.60)	−1.59	0.112
Female	135	21 (15.56%)			36.30 (35.60, 36.60)		
Multiple pregnancy							
Yes	240	11 (4.58%)	97.82	<0.001	36.20 (35.50, 36.60)	−2.28	0.20
No	74	39 (52.70%)			36.30 (36.00, 36.6)		
Caesarean section							
Yes	209	38 (18.19%)	2.38	0.12	36.30 (35.60, 36.60)	−2.68	0.007
No	105	12 (11.43%)			36.10 (35.40, 36.50)		
Invasive ventilation (medicine)							
Yes	72	30 (41.67%)	46.24	<0.001	36.50 (35.70, 37.40)	−3.35	0.001
No	242	20 (8.26%)			36.20 (35.50, 36.50)		
Duration of non-invasive ventilation							
Yes	136	42 (30.88%)	40.10	<0.001	36.40 (35.50, 37.18)	−2.24	0.025
No	178	8 (4.49%)			36.20 (35.58, 36.5)		
High flow through the nose							
Yes	91	31 (34.07%)	31.50	<0.001	36.20 (35.50, 37.40)	−1.07	0.28
No	223	19 (8.52%)			36.30 (35.60, 36.50)		

**Table 2:** Comparison of baseline between the two groups of children.

Sports event	Non-feeding disorder group (*n* = 264)	Feeding disorder group (*n* = 50)	Z-value/χ^2^ value	*P*-value
Gestational age at birth [M(Q1, Q3), weeks]				
≤28	28 (27.6, 28)	27.6 (26.1, 27.6)	−1.892	0.059
28^+1^–32	30.4 (29.5, 31.3)	29.6 (29.2, 30.35)	−1.878	0.060
32^+1^–34	33.2 (32.57, 33.52)	33.2 (32.4, 33.47)	−0.423	0.672
34^+1^–37	36.1 (35.5, 36.4)	36.4 (35.4, 36.50)	−1.227	0.220
Birth weight [M(Q1, Q3), kg]				
≤1	0.90 (0.90, 0.98)	0.87 (0.74, 0.94)	−1.695	0.090
1–1.5	1.31 (1.20, 1.42)	1.27 (1.15, 1.35)	−1.497	0.134
1.5< x <2.5	1.97 (1.75, 2.3)	1.77 (1.53, 2.02)	−2.161	0.031
>2.5	2.75 (2.60, 2.95)	2.75 (2.62, 3.01)	−0.126	0.900
Gender (cases)				
Male	150	29	−0.024	0.877
Female	114	21		
Multiple pregnancies (cases)				
Singleton	201	39	0.081	0.774
Multiple births	63	11		

### One-way logistic regression analysis

The children were categorized into a feeding-impaired group (*n* = 50) and a non-feeding-impaired group (*n* = 264) according to the presence or absence of a feeding disorder at 37 weeks of corrected gestational age. The results of univariate analysis showed that the preterm infants in both groups had the following characteristics in terms of birth gestational age, birth weight, Apgar score, invasive ventilation, duration of non-invasive ventilation, high-flow ventilation, duration of centralized oxygen, time of initiation of oral feedings, time of gastric tube removal, number of days of transition to feeding, absolute eosinophils, absolute eosinophilic granulocytes, absolute lymphocytes, absolute monocytes, erythrocyte count, leukocyte count, albumin, Comparisons in terms of total serum protein, calcium, and calcitoninogen showed statistically significant differences (*P* < 0.05) (Table 3).

**Table 3:** Unifactorial analysis of feeding disorders in preterm infants.

Sports event	Non-feeding disorder group (*n* = 264)	Feeding disorder group (*n* = 50)	t-value/Z-value	*P*-value
Gestational age at birth [M(Q1, Q3), weeks]	35.5 (32.23, 36.3)	32.4 (29.28, 36.05)	−3.37	0.001
Birth weight [M(Q1, Q3), kg]	2.55 (1.75, 2.78)	1.47 (1.23, 2.30)	−5.04	<0.001
1 min Apgar score [M(Q1, Q3), points]	10 (10, 10)	8 (6, 10)	−7.87	<0.001
5 min Apgar score [M(Q1, Q3), points]	10 (10, 10)	10 (9, 10)	−7.18	<0.001
10 min Apgar score [M(Q1, Q3), points]	10 (10, 10)	10 (9.75, 10)	−5.56	<0.001
Invasive ventilation [M(Q1, Q3), d]	0 (0, 0)	2 (0, 5)	−6.80	<0.001
Duration of noninvasive ventilation [M(Q1, Q3), d]	0 (0, 7)	11 (4, 28.5)	−6.56	<0.001
High flow [M(Q1, Q3), d]	0 (0, 0)	3.5 (0, 13)	−6.13	<0.001
Center oxygen [M(Q1, Q3), d]	0 (0, 4)	5 (2, 11)	−5.25	<0.001
Time to start oral feeding [M(Q1, Q3), weeks]	35.5 (34.23, 36.3)	35.55 (35.1, 36.5)	−2.10	0.035
Gastric tube removal time [M(Q1, Q3), weeks]	36.15 (35.5, 36.4)	37.55 (37.2, 38.23)	−10.94	<0.001
Feeding transition days [M(Q1, Q3), d]	0 (0, 8.75)	15 (8, 20.25)	−8.216	<0.001
Absolute basophil values [M(Q1, Q3), 10^9 ^/L]	0.03 (0.02, 0.07)	0.02 (0.01, 0.03)	−4.26	<0.001
Absolute eosinophil value [M(Q1, Q3), 10^9 ^/L]	0.21 (0.12, 0.34)	0.15 (0.07, 0.26)	−2.79	0.005
Absolute lymphocyte values [M(Q1, Q3), 10^9 ^/L]	3.13 (2.52, 3.93)	2.75 (1.86, 3.79)	−2.07	0.039
Absolute monocyte values [M(Q1, Q3), 10^9 ^/L]	1.05 (0.75, 1.43)	0.79 (0.51, 1.15)	−3.58	<0.001
Platelet count (x ± s, 10^9 ^/L)	272.68 ± 66.62	259.3 ± 76.28	−1.49	0.137
Erythrocyte count [M(Q1, Q3), 10^12 ^/L]	4.67 (4.22, 5.04)	4.26 (3.75, 4.64)	−3.85	<0.001
Leukocyte count [M(Q1, Q3), 10^9 ^/L]	11.26 (8.09, 14.08)	8.98 (6.04, 11.97)	−2.55	0.011
Albumin [M(Q1, Q3), g/L]	31 (29.1, 32.6)	29.05 (25.68, 31.18)	−4.12	<0.001
Direct bilirubin [M(Q1, Q3), umol/L]	9.35 (8.34, 10.3)	9.15 (8, 9.8)	−1.28	0.200
Indirect bilirubin [M(Q1, Q3), umol/L]	83.7 (67.9, 104.18)	83.35 (65.3, 97.45)	−0.30	0.766
Total serum protein [M(Q1, Q3), g/L]	45.7 (42.7, 49.6)	44.6 (38.45, 47.98)	−2.54	0.011
Calcium [M(Q1, Q3), mmol/L]	1.97 (1.81, 2.07)	1.83 (1.63, 1.99)	−3.49	<0.001
Calcitoninogen [M(Q1, Q3), ng/ml]	2.80 (0.93, 6.94)	4.15 (1.35, 12.98)	−2.01	0.044

### Multifactor logistic regression analysis

The presence of a feeding disorder at 37 weeks of corrected gestational age of the child was used as the dependent variable Y, and the 25 variables mentioned above were used as the independent variables X. The final prediction model incorporated seven clinical indicators as predictors: birth gestational age, birth weight, Apgar 1 min, time to noninvasive ventilation, absolute value of monocytes, leukocyte count, and calcium (Tables 4, 5).

**Table 4:** Assignment of independent variables in multifactor binary logistic regression analysis.

independent variable	Assignment of values
Gestational age at birth (weeks)	1 ≤ 28, 2 = 28^+1^–32, 3 = 32^+1^–34, 4 = 34^+1^–37
Birth weight (kg)	1 ≤ 1, 2 = 1–1.5, 3 = 1.5< × <2.5, 4 ≥ 2.5
1 min Apgar score (points)	Original value input
Duration of non-invasive ventilation (d)	Original value input
Absolute monocyte value (10^9 ^/L)	Original value input
White blood cell count (10^9 ^/L)	Original value input
Calcium (mmol/L)	Original value input

**Table 5:** Multifactorial logistic regression analysis of factors influencing feeding barriers in preterm infants.

Variant	Regression coefficient	Standard error	Wald *χ*^2^ value	*P*-value	OR value	95% confidence interval
Gestational age at birth	0.696	0.191	13.236	0	2.005	1.378–2.917
Birth weight	−1.688	0.658	6.574	0.01	0.185	0.051–0.672
1 min Apgar rating	−0.693	0.137	25.468	0	0.5	0.382–0.654
Duration of non-invasive ventilation	0.087	0.031	8.155	0.004	1.091	1.028–1.159
Absolute value of monocytes	−2.384	0.683	12.189	0	0.092	0.024–0.351
White blood cell count	0.2	0.064	9.866	0.002	1.221	1.078–1.384
Calcium (chemistry)	−2.216	0.938	5.576	0.018	0.109	0.017–0.686
Constant	−11.938	5.242	5.188	0.023	–	–

### Predictive modeling of feeding disorders in preterm infants

With the presence of feeding disorders at 37 weeks of corrected gestational age of the child as the dependent variable and the above seven indicators (birth gestational age, birth weight, Apgar 1 min, noninvasive ventilation time, absolute value of monocytes, leukocyte count, and calcium) as the independent variables, the regression equation was constructed as Logit (P) = −11.938 + 0.696 × birth gestational age—1.688 × birth weight—0.693 × 1 min Apgar score + 0.087 × duration of noninvasive ventilation—2.384 × absolute value of monocytes + 0.2 × leukocyte count—2.216 × calcium, and the predictive accuracy (accuracy, Acc) of this model was 91.401%, which indicates that the efficacy of the present model is better.The Hosmer-Lemeshow goodness-of-fit test showed that *χ*^2^ = 11.49, *P* = 0.175, *P* > 0.05, which indicates the goodness of fit of the model. The tolerance and variance inflation factor of the seven variables in the model were obtained through the linear regression function of SPSS25.0 statistical software(Table 6).The covariance diagnosis suggests that the tolerance of each variable is much greater than 0.1, and the variance inflation factor is less than 10, so it can be assumed that there is no multicollinearity among the variables.

**Table 6:** Predictor covariance analysis.

Variant	Tolerance	Variance inflation factor (VIF)
Gestational age at birth	0.102	9.851
Birth weight	0.140	7.135
Apgar 1 min	0.302	3.311
Non-invasive ventilation (NIV)	0.181	5.520
Absolute value of monocytes	0.803	1.246
White blood cell count	0.526	1.902
Calcium (chemistry)	0.414	2.418

### Comparison of ROC curves of indicators on feeding disorders in preterm infants

Based on the results of multifactorial binary logistic regression analysis, ROC curves were plotted and analyzed for the indicators that met *P* < 0.05 in the multifactorial analysis. The results showed that the AUC of birth weight for predicting feeding disorders was 0.725 (*P* < 0.001, 95% CI 0.642–0.808), with a maximum Yoden index of 0.407 and an optimal cut-off value of 1.555 kg, with a sensitivity of 60.0% and a specificity of 80.7%, and that the AUC of the Apgar 1-minute score for predicting feeding disorders was 0.787 (*P* < 0.001, 95% CI 0.709–0.865), with a maximum Jordon's index of 0.513, an optimal cutoff value of 9.5 points, a sensitivity of 74%, and a specificity of 77.3%; and the AUC for noninvasive ventilation time to predict feeding disorders was 0.765 (*P* < 0.001, 95% CI 0.694–0.836), with a maximum Jordon's index was 0.507, with an optimal cut-off value of 2.5 days, a sensitivity of 84%, and a specificity of 66.7%; suggesting that birth weight, 1-minute Apgar score, and noninvasive ventilation time all have some predictive ability for feeding disorders in preterm infants.

**Figure 1:**
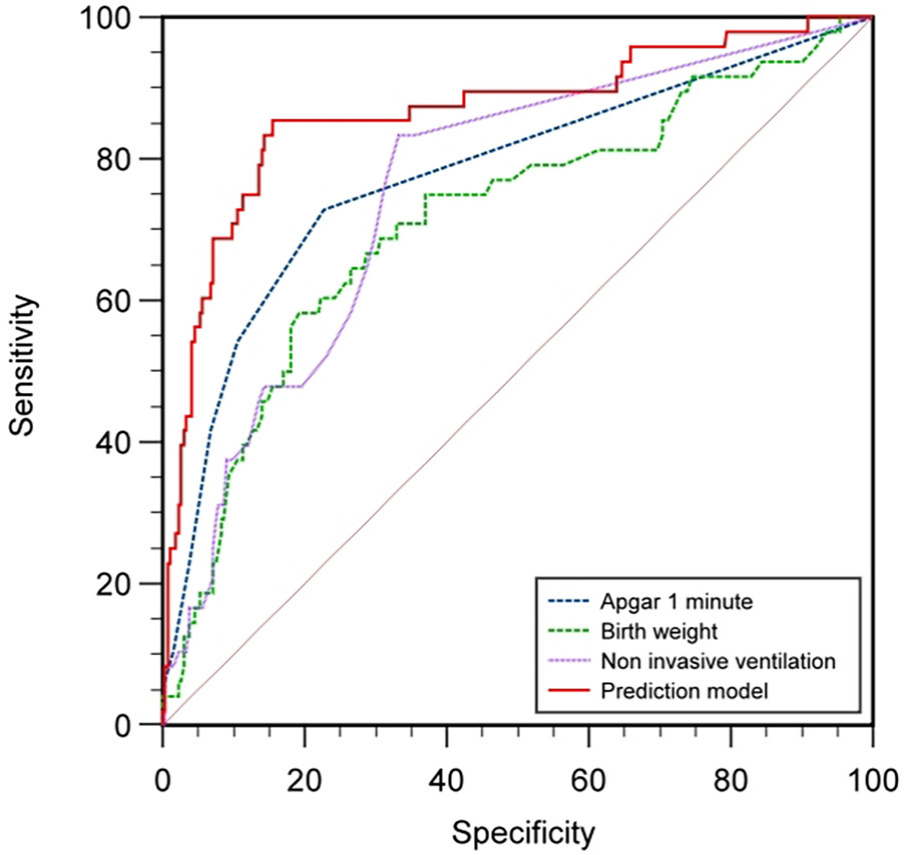
Comparison of ROC curves by indicator.

The area under the curve AUC for constructing a prediction model for feeding disorders to jointly predict feeding disorders was 0.866 (*P* < 0.001, 95% CI 0.801–0.932), which indicated that the prediction model had a better prediction ability for feeding disorders and its maximum Yoden index was 0.699, which at this time corresponded to a critical value of 0.169, with a sensitivity of 85.4% and a specificity of 84.5%. The results analyzed statistically showed that the AUC of the prediction model for predicting feeding disorders were all better than the single indicators of birth weight (Z = 3.584, *P* *<* 0.001), time of noninvasive ventilation (Z = 3.21, *P* = 0.001), and the Apgar 1-minute score (Z = 2.568, *P* = 0.010), and the difference was statistically significant (Table 7; Figure 1).

**Table 7:** Comparison of ROC curves and AUC values of indicators on feeding disorders in preterm infants.

Test variable	AUC	Maximum Jordon index	Truncation value	Standard error	95% CI	*P*-value	Sensitivity (%)	Specificity (%)	Accuracy (%)
Birth weight	0.725	0.407	1.555	0.042	0.642–0.808	<0.001	60.0%	80.7%	84.39%
1 min Apgar rating	0.787	0.513	9.500	0.040	0.709–0.865	<0.001	74%	77.3%	85.03%
Duration of non-invasive ventilation	0.765	0.507	2.500	0.036	0.694–0.836	<0.001	84%	66.7%	84.39%
Predictive probability	0.866	0.699	0.169	0.033	0.801–0.932	<0.001	85.4%	84.5%	91.40%

ROC Curves: Comparison between the combined model (red) and top individual predictors. Shaded areas represent 95% CIs from bootstrapping (1,000 iterations).

Threshold Markers: Optimal cutoff (0.65) balances sensitivity (85.4%) and specificity (84.5%) using Youden's index.

## Discussion

Transoral feeding involves precise coordination of oral motor functions, respiratory and digestive systems. Compared to full-term infants, preterm infants often have to face more survival challenges such as respiratory distress, respiratory distress syndrome, sepsis, oxygen desaturation or bradycardia due to their immature development (20). In this study, we found that 15.92% of preterm infants corrected for a gestational age of 37 weeks had significant feeding problems requiring gastric tube-assisted feeding, despite being close to the physiologic status of full-term infants. This finding emphasizes that even when preterm infants reach a stage of relative maturity, their feeding problems cannot be ignored and require more careful and individualized interventions by the healthcare team.

### Developmental maturity of preterm infants

Low birth gestational age is a risk factor for feeding disorders. The younger the gestational age, the higher the incidence of feeding disorders in preterm infants (21), susceptible to decreased oxygen saturation and apnea due to weak sucking, immature swallowing, and uncoordinated sucking-swallowing-breathing (22), and with low maturity of intestinal function, the higher the risk of FI. In this study, we found that the gestational age of the feeding disorder group was lower than that of the non-feeding disorder group (Z = −3.37, *P* = 0.001).

Low birth weight is a risk factor for feeding disorders in preterm infants. The higher the birth weight the more tolerant the child is and the more mature the organs are. At the same time, a heavier birth weight reduces feeding intolerance (FI) in preterm infants, suggesting better digestive function (23, 24). In the present study, we found that children in the feeding disorder group were lighter (*P* < 0.001) and the lower the weight, the greater the gestational age at which the gastrostomy tube was removed, suggesting that birth weight significantly affects feeding function in preterm infants.

### Organizational functioning of preterm infants

Low white blood cell (WBC) count is a risk factor for feeding disorders in preterm infants and is associated with birth gestational age, immune function, and inflammatory response (25). In preterm infants, WBC rises with increasing birth weight and peaks within the first day of life. Neonatal WBC at birth was (15–28) × 10^9 ^/L (26) and 32–34 weeks preterm WBC was (13.01 ± 5.18) × 10^9 ^/L (27), which is in agreement with the present study. The leukocyte erythrocyte count ratio (LER) correlates intrapulmonary lesions (28–30) as a risk factor for aspiration pneumonia (31), and low WBC also correlates with brain injury and NEC (32, 33), with blood redistribution during brain injury, gastrointestinal hypoxia-ischemia is the first to occurs, inhibiting gastrointestinal motility (34). And brain function, gastrointestinal function, and lung function affect swallowing function (35). The present study showed that WBC [8.98 (6.04, 11.97)] was lower in the feeding disorder group than in the non-feeding disorder group [11.26 (8.09, 14.08)] (*P* < 0.001), suggesting that low WBC may be related to feeding disorder.

Low absolute monocyte values are a risk factor for feeding disorders in preterm infants. Monocytes are involved in the body's defense processes (36). They are greatly altered when the organism undergoes inflammation or other diseases (37). Studies have shown that monocytes and other peripheral immune cells may affect brain function (38) and that absolute monocyte values are reduced in the early stages of the onset of neonatal NEC (39) and are lowest at the time of NEC diagnosis (40). In the present study, we found that the absolute monocyte values of children in the feeding disorder group were lower than those in the non-feeding disorder group, and we consider that there may be an indirect relationship between the low absolute monocyte values and the initial intestinal inflammation, which may affect the feeding function of preterm infants.

Low blood calcium values are a risk factor for feeding disorders in preterm infants. Blood calcium is important for metabolic and physiologic functions and is associated with maternal and own calcium and phosphorus regulation (41). The lower the gestational age at birth and the lower the birth weight, the lower the blood calcium (42), which is associated with a shorter calcium reserve time and insufficient parathyroid response in preterm infants (43); therefore, preterm infants generally have low blood calcium in the first 24–36 h of life, which is consistent with the present study. Extracellular Ca is involved in the excitability of neurons and muscle cells (44). Hypocalcemia negatively affects contractility and motility of the gastrointestinal tract (45). The blood calcium level in the feeding disorder group was [1.83 (1.63, 1.99) mmol/L], which was lower than that in the non-feeding disorder group [1.97 (1.81, 2.07) mmol/L] (*P* < 0.05), suggesting that low blood calcium is associated with insufficient excitability of swallowing muscles and gastrointestinal dysfunction in preterm infants.

### Extent of damage to preterm infants

A low Apgar score at 1 min after birth is a risk factor for feeding disorders in preterm infants. The score reflects the immediate health status of the preterm infant, with higher scores predicting healthier newborns and greater chances of survival (46). A low score may suggest that the child is at risk of hypoxia and asphyxia (47), which is strongly associated with secondary multi-organ damage (e.g., lung, gastrointestinal, brain) (48), which are critical for transoral feeding. The present study showed that 1-minute Apgar scores of children in the feeding-impaired group were lower than those in the non-feeding-impaired group (*P* < 0.001), suggesting that a former hypoxic state may be associated with feeding impairment in preterm infants.

Prolonged noninvasive ventilation is a risk factor for feeding disorders in preterm infants. For very preterm infants, respiratory disease is still the most important clinical problem because lung tissues are not yet mature (49), so mechanical ventilation is needed to assist respiration, but prolonged mechanical ventilation is prone to respiratory dependence, which inhibits spontaneous respiration to a certain degree (50) and affects the coordination of sucking, swallowing, and respiration (51), which results in the feeding process of the children This can lead to disorders of gas exchange, breath-holding, decreased oxygen saturation, and decreased heart rate (52), affecting feeding function and even aspiration (53). In addition, noninvasive ventilation increases gastrointestinal insufflation and intestinal wall dilatation, which reduces gastrointestinal blood flow and slows down gastrointestinal peristalsis (54), affecting gastrointestinal function, and the longer the duration of noninvasive ventilation, the more likely that feeding intolerance will occur (55), which will have a certain effect on transoral feeding. In the present study, we found that the duration of noninvasive ventilation was longer in the feeding-impaired group (*P* < 0.001), suggesting that poor lung function is associated with feeding impairment in preterm infants.

### ROC curve analysis of predictive models for prediction of feeding disorder outcomes in preterm infants

In this study, seven indicators were included in the prediction model, including “birth gestational age”, “duration of noninvasive ventilation”, “white blood cell count”, “birth weight”, “1-minute Apgar score”, “absolute monocyte value”, and “calcium”, which covered a wide range of clinical indicators of preterm infants. “1 min Apgar score”, “absolute value of monocytes”, and “blood calcium”, which cover a wide range of clinical indicators of preterm infants. Birth gestational age, birth weight and developmental maturity of preterm infants are related to each other, white blood cell count, absolute monocyte value, and blood calcium are related to the body function of preterm infants, and 1-minute Apgar score and noninvasive ventilation time are related to the degree of damage of preterm infants. In this study, the predictive model was found to be superior to single-indicator prediction with high sensitivity (85.4%) and specificity (84.5%), and a prediction accuracy of 91.4%.The sensitivity of 85.4% suggests that the model was able to identify more than 80% of the potentially feeding-disordered children, which is helpful in reducing the risk of underdiagnosis and in reducing delayed interventions. Children at high risk (positive model prediction) can be prioritized to receive bedside swallowing function assessment to shorten the diagnostic delay. The present predictive model integrates the developmental maturity, degree of organic function and impairment, and organic function of preterm infants, which facilitates better prediction of the prognosis of feeding disorders and is consistent with our hypothesis.

## Conclusion

In this study, low birth gestational age, low birth weight, low white blood cell count, low absolute monocyte value, low blood calcium value, low Apgar score at 1 min after birth, and prolonged noninvasive ventilation were found to be risk factors for feeding disorders in preterm infants. This prediction model has high sensitivity, specificity, and predictive accuracy for prediction of feeding disorders in preterm infants, which can warn preterm infants of the risk of developing feeding disorders, and help clinicians to identify this group of infants and take active rehabilitative therapeutic interventions to improve the feeding function, which can provide clinical value for the early removal of gastrostomy tubes and shorten the length of hospitalization.

## Limitations

The main limitation that limits this study is its retrospective design. Other limitations: Model validation was not performed. Potential confounding variables that may affect the outcome, such as maternal health status, antenatal care, initiation of breastfeeding and socioeconomic factors were not considered, and multicenter validation was lacking.

## Data Availability

The raw data supporting the conclusions of this article will be made available by the authors, without undue reservation.
